# Transcutaneous auricular vagus nerve stimulation for postoperative nausea and vomiting in gynecological laparoscopic surgery: a randomized controlled trial protocol

**DOI:** 10.1080/07853890.2026.2696599

**Published:** 2026-07-11

**Authors:** Peilin Li, Lijun Fan, Qingcai Chen, Xiaoming Jia, Guowang Yang, Guangchao Zhang

**Affiliations:** a Department of Anesthesiology, The First Affiliated Hospital of Soochow University, Suzhou, China; b Institute of Anesthesiology, Soochow University, Suzhou, China

**Keywords:** Transcutaneous auricular vagus nerve stimulation, postoperative nausea and vomiting, gynecological laparoscopic surgery

## Abstract

**Introduction:**

Postoperative nausea and vomiting (PONV) are common adverse events following gynecological laparoscopic surgery. This study aims to evaluate the efficacy of transcutaneous auricular vagus nerve stimulation in reducing the incidence of PONV within 24 h after gynecological laparoscopic surgery.

**Methods and analysis:**

This single-center, prospective, double-blinded, randomized and sham-controlled trial will enroll 200 patients scheduled for gynecological laparoscopic surgery. Participants will be randomized in a 1:1 ratio to either the taVNS group or the sham group. The taVNS group will receive electrical stimulation lasting for 30 min after anesthesia induction. Sham group will not provide stimulation. The primary endpoint is the incidence of PONV within the first 24 h after surgery. Secondary endpoints include the severity of PONV, the usage of rescue antiemetics and 5-point Likert satisfaction scale. Exploratory endpoints include consumption of opioids and heart rate variability within 24 h after surgery.

**Discussion:**

The results of study will provide key evidence on the efficacy and safety of intraoperative taVNS for preventing PONV after gynecologic laparoscopic surgery.

Trial registration: Chinese Clinical Trial Registry (registration No. ChiCTR2600120159; registered on March 10, 2026)

## Introduction

Postoperative nausea and vomiting (PONV) are common adverse events after surgery and anesthesia, with an increased risk of reflux aspiration, suture dehiscence, postoperative bleeding, electrolyte imbalance, prolonged hospital stay and reduced patient satisfaction [1,2]. Independent risk factors most commonly associated with PONV include female sex, non-smoker, history of PONV and postoperative opioids [1,3]. Meanwhile, pneumoperitoneum, use of inhalation anesthetics and intraoperative opioid analgesics also increase the risk of PONV [4]. Patients undergoing gynecological laparoscopic surgery belong to the PONV high-risk population, with the incidence rates of moderate to severe PONV remaining around 30%–40%[4,5].

The underlying neural circuits associated with PONV include input *via* the vagus nerve, neurons in the area postrema, the vestibular system, and potentially other cell types [6,7]. Meanwhile, some exogenous stimuli could induce PONV by releasing emetic neurotransmitters to activate corresponding emetic receptors, such as those for 5-hydroxytryptamine (5-HT), neurokinin-1 (NK-1R), dopamine D2 and D3, mu and kappa opioid, muscarinic M1, and histamine H1 receptors [8,9]. Currently, the prevention of PONV mainly involves antiemetic drug intervention by acting on receptors. However, most of the antiemetics can have adverse effects and the effectiveness can be inconsistent among patients [10]. The multiple therapeutic approaches of PONV should be explored.

Transcutaneous auricular vagus nerve stimulation (taVNS) is an emerging neuromodulatory technique, which has been explored to regulate central and peripheral organs function [11–13]. The external ear is the only place on the body where VN sends its peripheral branch [14,15]. The stimulation on the auricular branch of the vagus nerve (ABVN) could connect directly to the brainstem, and then to higher brain regions *via* extensive projections to second and third order neurons within the brain [16,17]. Consequently, given that taVNS involves both sympathetic and parasympathetic branches, it generates systemic effect. It is reported taVNS regulates the gastrointestinal tract in rats with functional dyspepsia by increasing the gastric emptying rate improving gastric motility, reducing gastric sensitivity, and alleviating low-grade inflammation [18]. Moreover, more researchers focus on the effect of taVNS on postoperative pain management or perioperative anxiety [19,20]. However, there is limited research on impact of taVNS on PONV.

Therefore, this double-blind randomized controlled trial will be designed to investigate the effect of the taVNS on the incidence of PONV within 24 h after surgery in female patients undergoing elective gynecologic laparoscopic surgery under general anesthesia. As a secondary aim, we plan to assess the effects of taVNS on the severity of postoperative nausea, the dosage of antiemetic drugs administered, and patient satisfaction.

## Patients and methods

### Study design and registration

**Figure 1.**
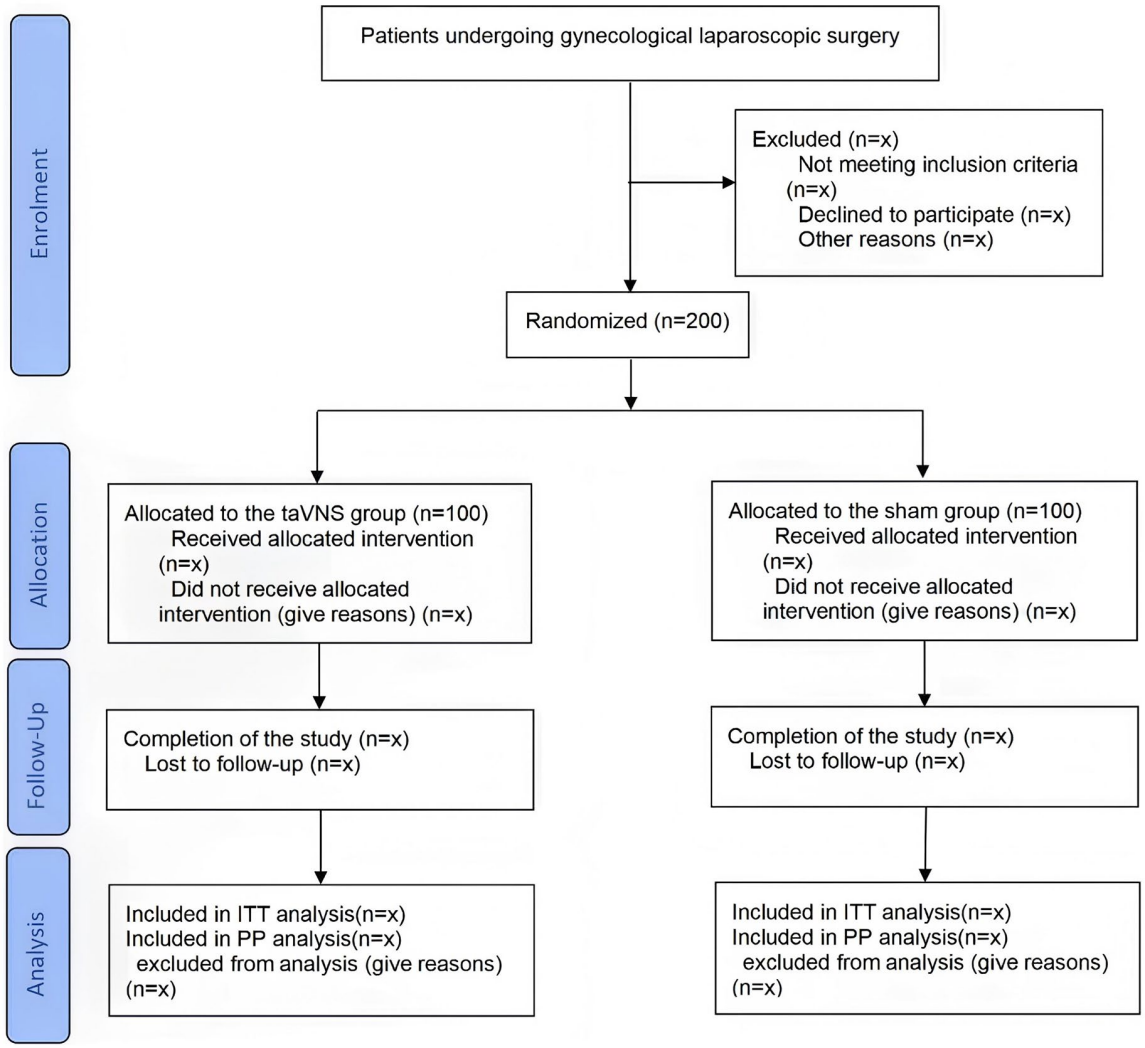
Flow diagram of this trial. taVNS: transcutaneous auricular vagus nerve stimulation; ITT: intention-to-treat; PP, per-protocol.

This study is a single-center, prospective, double-blinded, randomized and sham-controlled clinical trial. The trial has been approved by the Ethics Committee of the First Affiliated Hospital of Soochow University (Approval no.2025-1298) and registered at clinical trials.gov (registration No. ChiCTR2600120159). The trial is conducted in accordance with the Declaration of Helsinki and will be monitored by the clinical trial center of the hospital. Written informed consent will be obtained from each patient before randomization and surgery. The SPIRIT checklist is included in supplemental file S1. The trial flow diagram is shown in Figure 1. Schedule of subject enrollment, interventions, and outcome measurements are presented in Table 1.

**Table 1.** Schedule of subject enrollment, interventions, and outcome measurements.

	Study period							
	Enrollment	Allocation	Post-allocation					Close-out
TIMEPOINT	Preanaesthesia visit	preoperative	Intraoperative	1 h	2 h	6 h	12 h	24 h
**Enrollment**	x							
Eligibility screen	x							
Informed consent	x							
Baseline assessment	x							
Randomization		x						
**Inventions**								
The taVNS group			x					
The sham group			x					
**Assessments**								
Nausea				x	x	x	x	x
Vomiting				x	x	x	x	x
NRS				x	x	x	x	x
Rescue antiemetics								x
5-point Likert scale								x
Consumption of opioids								x
Heart rate variability		x	x					x
Adverse events			x	x	x	x	x	x

taVNS: transcutaneous auricular vagus nerve stimulation; NRS: numerical rating scale.

### Study population

Patients scheduled for gynecological laparoscopic surgery in the First Affiliated Hospital of Soochow University will be recruited for the research. Patients will be eligible for participation if they are aged from 18 to 65 and have an American Society of Anesthesiologists physical status of class I to II. Exclusion criteria include: (1) infection, malformation or injury in the outer ear; (2) administration of antiemetic drugs, glucocorticoids or opioids within 24 h preoperatively; (3) neurologic, or cognitive dysfunction, or psychological disorders; (4) pregnancy or breastfeeding; (5) refusing to provide informed consent.

The preparatory phase of this study (including staff training, equipment calibration and finalization of standard operating procedures) commenced on January 24, 2026 and the first participant was enrolled on March 16, 2026, which was shortly after the completion of trial registration. At the time of submitting this manuscript revision, patient recruitment is ongoing and has not been completed. Those enrolled participants had no history of chronic nausea and vomiting, no history of motion sickness, and no plan to use opioid drugs for analgesia within 24 h after surgery.

### Randomization and blinding

After informed consent is obtained, block randomization is employed. An independent administrator not involved in this study sets a block length of 4, generating a random sequence using the online Sealed Envelope tool. Participants are randomized in a 1:1 ratio to either the taVNS group or the sham group. During this process, allocation concealment is guaranteed by using sealed envelopes and the anesthesiologist responsible for the surgery has no a priori knowledge of the intervention assignment. The medical team providing care during the perioperative period, outcome assessors, investigators, and patients are also blinded to the group assignment. After the participant enters the operating room, an anesthesia assistant who is not involved in the data collection and outcome assessment opens the envelope in the absence of the anesthesiologist, assigns the patient to either the taVNS or the sham group, sets the vagus nerve electrical stimulation parameters, and places the vagus nerve electrical stimulation device into an opaque black bag. The anesthesiologist then performs anesthesia induction immediately. Due to the anterograde amnesic effect of anesthesia, the patient remains unaware of the intervention postoperatively. To ensure the effectiveness of patient blinding, a random selection of patients is surveyed before unblinding to determine which group they believed they had been assigned to. The proportion is then calculated, and if it is close to 1:1, it indicates successful blinding. Follow-up personnel and ward staff remain unaware of the group assignments. After statistical results are generated, unblinding is performed for all participants.

### Anesthesia management

All patients are given Tracheal Intubation General Anesthesia. Premedication is not permitted in either group, including anticholinergic drugs or sedatives. After entering the operating room, venous access is established, if necessary, radial artery catheterization is performed. Then, multi-functional monitor is connected. Non-invasive blood pressure (or invasive arterial blood pressure), blood oxygen saturation, electrocardiography, PETCO2, and depth of anesthesia are measured. Anesthesia is induced with propofol (1.5–2.5 mg/kg) and sufentanil (0.3-0.5ug/kg). A low dose of Rocuronium (0.8 mg/kg) is administered to facilitate intubation. After tracheal intubation, mechanical ventilation with 60% oxygen in air is applied and adjusted to achieve the end-tidal carbon dioxide partial pressure between 35 to 45 mmHg. Anesthesia is maintained with end-tidal concentration of sevoflurane (1%-2.5%), plus a continuous infusion of refentanil (5–10 ug · kg − 1 · *h* − 1), and cisatracurium 0.3 mg/kg per hour. During the operation, the infusion rates of propofol and refentanil are adjusted to maintain hemodynamic stability. All participants in both groups will receive standardized multimodal antiemetic prophylaxis consisting of 5 mg dexamethasone (administered at anaesthesia induction) and 8 mg ondansetron (administered before the end of surgery). Supplemental postoperative analgesia is provided at the discretion of attending anesthesiologist or patient and could include opioids, nonsteroidal anti-inflammatory drugs, and other analgesics.

### Study intervention

Before anesthesia, an anesthesia assistant not involved in data collection will open the opaque envelopes containing randomized sequences and then adjust the parameters of the taVNS device. In both groups, the electrodes will be placed on the concha of the left ear and electrical stimulation will be delivered identically (pulse width, 200 microseconds; frequency, 20 Hz). The current intensity will be increased for 30 s until the participant reports a tingling sensation. Then the intensity will be decreased to a comfort level slightly below the above threshold. Following induction of anesthesia, the taVNS group patients will receive electrical stimulation lasting for 30 min according to the predefined parameters. In sham group, patients will not receive electrical stimulation by keeping device remain off for 30 min to maintain the sham state. Based on the anatomical evidence that the right ABVN innervates the sinoatrial node and may exert adverse effects on heart rate, electrical stimulation will be delivered *via* electrodes on the left concha of the ear in all participants [21]. During the intervention period, patients will be closely monitored for ECG, blood pressure, and pulse oxygen saturation (SpO_2_). Once severe bradycardia, high-degree AV block, or significant hypotension occurs, the stimulation will be immediately terminated and the following conditions will be considered as dropout cases, and the reason and date of dropout will be recorded in a case report form.

### Outcome

The primary endpoint is the incidence of postoperative nausea and vomiting (PONV) within the first 24 h after surgery. PONV is defined as the occurrence of any episode of nausea (an unpleasant subjective sensation associated with the urge to vomit) and/or vomiting (the forceful expulsion of gastric contents) during this period. The incidence will be assessed by active inquiry and documentation of the occurrence of these symptoms at 1, 2, 6, 12, and 24 h postoperatively. The primary outcome measure will be the proportion of patients experiencing at least one episode of nausea or vomiting within the first 24 h postoperatively.

Secondary endpoints are as follows: the severity of nausea or/and vomiting at 1, 2, 6, 12, and 24 h postoperatively, assessed using a 0–10 numerical rating scale (NRS), which is a patient self-assessment tool involves drawing a 10 cm line on paper, with 0 indicating no symptoms of nausea and 10 indicating excruciating severe vomiting; the types and dosage of drugs used for rescue antiemetic therapy within 24 h after surgery; and a 5-point Likert satisfaction scale with taVNS for the management of nausea and vomiting, with 1 to 5 points indicating ‘strongly disagree’ to ‘strongly agree’ respectively (supplemental file S2). In addition, consumption of opioids within 24 h after surgery converted to intravenous morphine milligram equivalents (MME); the autonomic nervous function assessed *via* heart rate variability (HRV) parameters (low-frequency/high-frequency ratio) at baseline (pre-induction), at the end of surgery, and at 24 h postoperatively, as well as the treatment effect of taVNS on the primary endpoint (PONV incidence) across predefined patient subgroups based on age strata and Apfel score categories are further explored.

### Data collection

Data will be collected using dedicated case report forms (CRFs) and managed in a secure electronic database. The demographic and baseline data include age, height, weight, BMI, ASA physical status, Apfel risk score, smoking status, history of motion sickness/PONV, comorbidities, and preoperative medications. Intraoperative data include anesthetic doses (propofol, remifentanil, volatile agents), vital signs at key time points, episodes of hypotension/bradycardia with interventions, surgical duration, fluid administration, and taVNS/Sham protocol details (group assignment, stimulation parameters, duration). Postoperative data include incidence of PONV assessed at 1 h after PACU arrival and 2, 6, 12, and 24 h postoperatively, nausea severity (11-point NRS) at the above time points, use and dose of rescue antiemetics within 24 h, and patient satisfaction (5-point Likert scale) at 24 h. Additional data collected include all adverse events (e.g. skin erythema, dizziness) with details on their nature, severity, and relationship to the intervention; heart rate variability; 24-hour postoperative opioid consumption; the total cumulative consumption of opioid analgesics; and blinding assessment (patient guess).

### Sample size

The sample size was calculated with the PASS 15.0 software. It is reported the incidence of PONV among Chinese patients undergoing elective gynecological laparoscopic surgery is approximately 40% following dual-agent prophylaxis with dexamethasone and ondansetron [5]. Based on previous studies, we assume that the incidence of PONV in the taVNS group would be reduced to 20%[22,23]. To provide a significance level of 0.05 and a power of 0.8, a sample size of 158 patients will be required. Considering potential protocol deviations and withdrawal of consent, the recruitment target will be increased to 100 patients in each group.

### Statistical plan

Two members of the research team will perform study consolidation and analysis. All statistical plans will be finalized prior to unblinding. Categorical data will be expressed as frequencies and percentages and continuous variables will be expressed as means and standard deviations for normally distributed variables or otherwise as medians and interquartile range. The Kolmogorov–Smirnov test will be used to detect the normality of distribution. The statistical analysis will be conducted using R software (version 4.3.1 or higher) and SAS software (version 9.4 or higher).

Patient demographic and baseline characteristics will be summarized using descriptive statistics for each group. No hypothesis testing will be performed for between-group comparisons at baseline. The balance between the taVNS and the sham group will be evaluated by calculating standardized mean differences (SMDs), with an SMD > 0.10 indicating substantial imbalance.

The primary endpoint, the incidence of PONV within 24 h postoperatively, will be analyzed in both the intention-to-treat (ITT) population (all randomized patients) and the per-protocol (PP) population (excluding patients with major protocol deviations, such as failure to apply stimulation, incorrect stimulation parameters, or surgical conversion). Between-group comparisons for the primary endpoint will be performed using the Chi-square test or Fisher’s exact test, as appropriate. The treatment effect will be reported as relative risk (RR) with a 95% confidence interval (CI). A multivariable logistic regression model will be fitted to adjust for potential confounding factors (baseline covariates with SMD > 0.10), with results presented as an adjusted odds ratio (OR) with 95% CI. Missing data for the primary endpoint will be handled under the missing-at-random assumption using multiple imputation.

All secondary endpoints will be analyzed in the ITT population only. The severity of postoperative nausea (NRS scores) will be compared between groups using the independent t-test or Mann-Whitney U test, with the effect size reported as the difference in means or medians with 95% CI. The use of rescue antiemetics and patient satisfaction scores will be compared using the Chi-square test (or Fisher’s exact test) and the Mann-Whitney U test, respectively. A Bonferroni correction will be applied for the three secondary endpoint comparisons, setting the significance level at *p* < 0.017 (0.05/3).

Exploratory analyses (e.g. heart rate variability parameters, postoperative opioid consumption) will be conducted in the ITT population without correcting for multiple comparisons. Effect estimates with 95% CIs will be reported for these endpoints, but P-values will not be emphasized. Subgroup analyses of the primary endpoint will be performed based on predefined factors such as age strata and Apfel score categories. Interaction terms will be tested in regression models to assess effect modification.

Safety data, including the incidence of adverse events, will be summarized descriptively by treatment group.

## Discussion

The study employed a randomized, double-blind, and sham-controlled design. This clinical study will group 200 gynecological patients who undergo laparoscopic surgery, and they will respectively receive left auricular vestibular vagus nerve electrical stimulation and sham stimulation. The primary endpoint is whether the incidence of postoperative nausea and vomiting (PONV) can be reduced. The secondary outcomes include determining whether the severity of nausea can be alleviated, reducing the dosage of antiemetic drugs, and improving patients’ satisfaction. The exploratory endpoints mainly include heart rate variability during the operation and the use of opioid drugs within 24 h after the operation. We will complete this trial in accordance with the Consolidated Standards of Reporting Trials guidelines.

PONV is a pivotal factor affecting the quality of postoperative recovery. taVNS represents a promising non-pharmacological intervention in PONV prevention [11,24,25]. The study employed a randomized, double-blind, and sham-controlled design to verify its effectiveness. Such a design minimizes bias and yields high-quality evidence. The intervention is administered intraoperatively, targeting a critical window within the neural pathways central to PONV pathogenesis.

If the study yields positive findings demonstrating a significant reduction in PONV incidence with taVNS, substantial clinical implications will be identified. First, the magnitude of PONV reduction achieved by taVNS should be compared with other established or emerging strategies, such as combined antiemetic regimens. This would establish its role in a multimodal PONV management strategy. Second, observed reductions in nausea severity and rescue antiemetic consumption have direct clinical relevance, as they confer potential advantages regarding patient comfort and alignment with enhanced recovery after surgery (ERAS) pathways. Detailed reporting of intraoperative anesthetic parameters including opioid and propofol doses provides essential context for interpreting PONV outcomes and enables comparison with other studies using comparable protocols.

The individualized current titration protocol based on the comfort sensory threshold before anesthesia induction is essential to ensure patients’ comfort and achieve consistent neurophysiological effects among patients [26]. Compared to multi-session protocols such as pre-, intra-, and postoperative protocols, the single intraoperative stimulation regimen presents distinct advantages and limitations concerning efficacy and clinical feasibility, meriting further discussion. Intraoperative stimulation targets the key window of PONV pathogenesis under anesthesia. It allows precise control and standardized delivery, unlike patient-dependent pre/post-op sessions. This pragmatic approach simplifies clinical implementation and trial design. The intraoperative timing of stimulation, while pragmatic, presents inherent limitations. First, its restricted window may not address preoperative anxiety or later-onset PONV occurring beyond 24 h. Second, the depth of general anesthesia could potentially attenuate the targeted neural response, possibly diminishing the intervention’s effect. Finally, this design does not allow us to explore whether a preemptive or multi-session strategy might yield superior outcomes. Moreover, maintaining successful blinding is paramount to minimizing bias in subjective outcomes, such as nausea scores and patient satisfaction; consequently, the blinding assessment employed in this study design will provide valuable insights for future.

Previous studies have shown that only mild and transient adverse events, such as erythema at the electrode site have been reported with taVNS [27]. In contrast, common antiemetic drugs are associated with side effects such as metoclopramide-induced oculogyric crisis [28], as well as potential sedation, headache, and QT interval prolongation linked to 5-HT3 receptor antagonists [29–31]. This highlights the potential advantage of taVNS in reducing polypharmacy and related drug side-effects. At present, no potential risks associated with left ear vagus nerve stimulation such as bradycardia and hypotension have been observed, likely due to the use of low-intensity transcutaneous auricular stimulation rather than invasive cervical vagus nerve stimulation. Additionally, theoretically, the right vagus nerve mainly controls the sinoatrial node [32]. Therefore, the possibility of experiencing bradycardia and hypotension due to stimulation of the right vagus nerve is greater. Furthermore, we collect patient-reported outcomes, including postoperative satisfaction. Exploratory analyses also detect the influence on opioid consumption of taVNS. These explorations require cautious interpretation, provide a more comprehensive assessment of the intervention’s utility and its impact from the patient’s perspective. Finally, preliminary subgroup analyses exploring whether the treatment effect is consistent across different ages or Apfel risk categories will need confirmation in future, larger trials.

This study is subject to several limitations. The findings of the single-center study require verification through multi-center studies with larger sample sizes. The relatively short follow-up period (24 h) may not fully capture late-onset PONV or potential sustained effects of taVNS. A single intraoperative stimulation may be insufficient, especially during prolonged surgeries, increasing the risk of a negative result. We plan subgroup analyses based on surgical duration. The results of this subgroup analysis may inform the design of future trials investigating optimized stimulation protocols. Despite the application of blinding, the distinct sensory perception induced by taVNS may compromise the blind in some patients or assessors. To verify the integrity of the blinding procedure, we will assess the final distribution of participants across study groups. A ratio close to 1:1 would provide indirect evidence of the validity of the outcome assessment under blinded conditions.

In conclusion, this study will provide key evidence on the efficacy and safety of intraoperative taVNS as a non-pharmacological and minimally invasive strategy for preventing PONV after gynecologic laparoscopic surgery. Positive results could support the application of taVNS in PONV management. It offers a novel, well-tolerated treatment option for patients. Furthermore, these results would establish a foundation for future research aimed at elucidating its mechanisms and evaluating its utility in diverse clinical scenarios.

## Supplementary Material

supplemental file S2 5point Likert Satisfaction Scale.docx

SPIRIT checklist.docx

## Data Availability

The full protocol, participant-level dataset, statistical plan, and informed consent materials can be available *via* contacting the corresponding author after the formal publication of this trial.
