# Supplementary material for: Transcutaneous auricular vagus nerve stimulation for postoperative nausea and vomiting in gynecological laparoscopic surgery: a randomized controlled trial protocol
Source: Ann Med. 2026 Jul 11;58(1):2696599. doi: 10.1080/07853890.2026.2696599 (PMC13371485; doi:10.1080/07853890.2026.2696599)
Supplement: supplemental file S2 5point Likert Satisfaction Scale.docx [file IANN_A_2696599_SM0822.docx]

**5-point Likert Satisfaction Scale**

| **Statement** | **Strongly Disagree** | **Disagree** | **Neutral** | **agree** | **Strongly agree** |
| --- | --- | --- | --- | --- | --- |
| Overall, the PONV prophylactic measure was comfortable |  |  |  |  |  |
| The treatment process did not cause me significant discomfort (e.g., pain, burning sensation) |  |  |  |  |  |
| This treatment effectively reduced my postoperative nausea and vomiting |  |  |  |  |  |
| Compared with other antiemetic methods I used, I prefer this non-invasive stimulation therapy |  |  |  |  |  |
| If I undergo similar surgery in the future, I would willingly to accept this treatment again |  |  |  |  |  |
